# Functional and Bioactive Properties of Fermented Microalgae and Their Biomass for Health Applications

**DOI:** 10.3390/molecules31111785

**Published:** 2026-05-22

**Authors:** Akif Emre Kavak, Enes Dertli

**Affiliations:** 1 Department of Bioengineering, Faculty of Chemical and Metallurgical Engineering, Yıldız Technical University, Istanbul 34349, Turkey; 2 Nuvita Biosearch R&D Center, Istanbul 34522, Turkey; 3 Department of Food Engineering, Faculty of Chemical and Metallurgical Engineering, Istanbul Technical University, Istanbul 34467, Turkey; enesdertli@itu.edu.tr

**Keywords:** lactic acid bacteria, microalgae, fermentation, fermented foods, bioactive compounds

## Abstract

In recent times, the importance given to versatile functional nutrition has increased, escalating interest in fermented foods and their potential health benefits. Fermentation is an ancient method frequently used to develop functional and bioactive products. Fermented microalgae and their biomass are important sustainable biotechnological resources for increasing the nutritional value, healthiness, and functionality of foods and for producing high-value-added bioactive compounds. The fermentation of microalgae encompasses the conversion of carbohydrates into sugar or organic substances by a range of microorganisms, particularly lactic acid bacteria (LAB). The fermentation process can activate numerous beneficial mechanisms by enhancing the bioavailability of bioactive compounds in microalgae. Lactic acid bacteria are widely used in food fermentation due to their safety and metabolic versatility. Their ability to produce organic acids, enzymes, and bioactive metabolites makes them suitable for modifying microalgal biomass. This review aims to provide a detailed and critical evaluation of fermented microalgae, including health effects, functional enhancements, bioactivities, and industrial applications.

## 1. Introduction

Nowadays, the relationship between food and health is paramount, and the demand for functional foods is increasing exponentially. People place great importance on their diets in order to live healthier and longer lives [1]. In this vein, interest in food supplements, functional food ingredients, and fermented foods is steadily increasing. The size of the food supplement market and that of the functional food/beverage market have been reported to reach approximately USD 210 billion and USD 374 billion, respectively, in 2025 [2]. With changing global conditions, producing sustainable food ingredients has become pivotal [3]. One of the most effective ways to produce sustainable food ingredients is through fermentation processes. Although fermentation has a very long history, it is still a widely used technology today [4,5]. It is also preferred because it ensures sustainable production and dynamic process conditions. Biochemical changes carried out by microorganisms in the fermentation process directly contribute to the release or alteration of nutrient components in the food matrix and also affect their functional, rheological and sensory properties. Fermented products can include bacteria pellets, metabolites or bioactive compounds secreted in the fermentation liquid [6,7]. The fermentation process can enhance the health effects of functional foods. Functional fermented foods possess numerous health benefits, including antioxidant, antimicrobial, and dietary advantages. Current trends towards functional fermented foods are increasing and will likely continue into the coming years [8,9].

Recently, microalgae have come to the forefront as a sustainable resource. Microalgae are an important biomass source with the potential for contributing to developing sustainable food systems and innovative transformation [10]. From the perspective of green technology and environmentally friendly production, microalgae are known for their ability to be grown on non-arable lands, their resistance to a wide variety of physical conditions, and their high production rate per square meter compared to plants [11]. Microalgae are unicellular photosynthetic organisms characterized by rapid growth and high biomass productivity. Microalgae are able to manufacture many bioproducts, including lipids, proteins, vitamins, carotenoids, and bioactive molecules [12,13,14].

Lactic acid bacteria (LAB) are a group of beneficial microorganisms known for their ability to convert sugars into lactic acid through fermentation [15]. As indispensable fermentation bacteria, LAB have GRAS (Generally Recognized As Safe) status and are considered important probiotics (FAO/WHO, 2002) [16]. LAB are among the major groups of bacteria of industrial importance and are used in food production, health regulation, and the production of macromolecules, metabolites and enzymes [17]. They are used in many fields, from probiotics and enzymes to animal nutrition and human health [18]. Given their probiotic properties, LAB are frequently associated with gut health and immune function [19]. LAB are abundant in fermented foods and have been a part of human life since ancient times. The beneficial effects of traditional fermented foods containing LAB on human health have been demonstrated for many years [20]. Some of these benefits are related to protein-derived bioactive products. They possess strong proteolytic activities that are highly important in the enzymatic modification of fermented foods [21]. Protein-derived products produced by LAB include ribosomally produced protein hydrolysate by-products used as natural preservatives and nutraceuticals. These protein-derived products, with their diverse application areas, are attracting industrial interest [22]. The first bioprocessing studies between LAB and microalgae began in the 1990s [23]. LAB found on the surface of microalgae interact with sugars released during degradation and other organic compounds [24]. They can also use microalgae such as spirulina, which contain high amounts of nitrogen molecules and protein, as a nitrogen source. Microalgae stand out as an important substrate for fermentation due to their high nutritional value. Recent research has shown that fermented algae products exhibit positive effects in terms of antioxidant, antimicrobial, flavor, texture and potential health effects [25,26].

Although the use of microalgae as biomass is widespread, the vast majority of current studies have focused separately on more basic applications in the food, agriculture and cosmetics industries. This review presents the effects of enriching the nutritional value of microalgae through fermentation on a healthy diet and developing functional foods, offering new perspectives for scientific research and industrial applications. Furthermore, it provides a comprehensive overview of various aspects of fermented microalgae, including applications in health, functional product development, and technological innovation.

## 2. Microalgae and Properties

Microalgae are single-celled, microscopic, photosynthetic microorganisms. Because of their photosynthetic ability, they can absorb H_2_O and CO_2_ and convert them into various forms of organic compounds with the help of sunlight. While most microalgae are eukaryotic, some are prokaryotic [27]. These microorganisms can be found in all aquatic environments. They can survive in freshwater (ponds, canals, puddles, and lakes), seas, and even very salty waters if suitable conditions are provided [28]. Microalgae have very high biodiversity, and it is known that there are approximately 200,000 microalgae species in the world. To date, it is estimated that around 50,000 species have been identified [29]. The way microalgae utilize sunlight and CO_2_ is similar to that of terrestrial plants. However, compared to terrestrial plants, microalgae reproduce by dividing within hours and can produce crops throughout the year, resulting in higher yields. Microalgae species absorb carbon dioxide from the atmosphere during the daytime period like a large generator and produce oxygen, which is vital for the life of humans and other living things [30]. The high commercial significance of the valuable metabolites that microalgae species accumulate cellularly, in addition to the biomass obtained from them and the availability of some species in environmental applications, further increases the current interest in microalgae and makes them an area of deep research in biotechnology. These organisms can naturally accumulate high amounts of proteins, pigments, fatty acids, vitamins, hydrocarbons, polysaccharides, and many other metabolites within their cells [31,32]. Microalgae can be produced in open pond systems or closed photobioreactor, tank, bubble, or bag systems. Microalgae-based products are used in many fields, such as food, cosmetics, pharmaceuticals, agriculture, farming, and environmental technologies [33]. On the other hand, the low product yields from microalgae and high cost of microalgal cultivation and purification restrict the economic feasibility of industrial production microalgae [34]. Additionally, microalgae are limited by bioavailability challenges. There are various intrinsic and extrinsic factors that jeopardize nutrient release and absorption; cell wall structures and nutrient solubility are among these. Thus, more research is needed to mitigate the relative disadvantages of microalgae and integrate them into more commercial applications [35,36].

**Figure 1:**
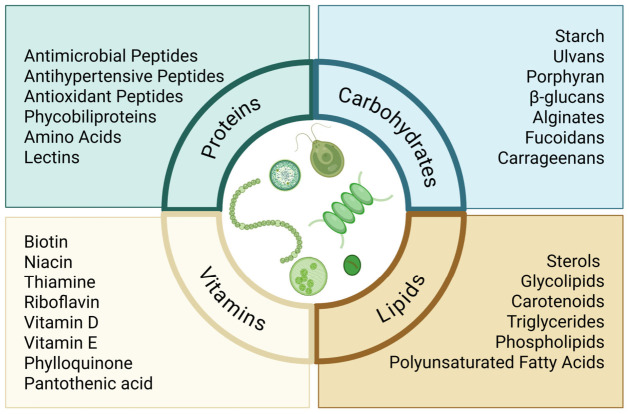
Metabolites produced by microalgae.

Microalgae are characterized by a high protein content (30–70% dry weight), depending on the species and cultivation conditions. These proteins often contain all essential amino acids, making them comparable to conventional protein sources such as soy and eggs [37]. Lipid content varies widely but may include significant levels of omega-3 fatty acids such as eicosapentaenoic acid (EPA) and docosahexaenoic acid (DHA) [38]. Microalgae are rich in vitamins (B-complex, vitamin E, vitamin K) and minerals (iron, calcium, magnesium) [39]. They also contain pigments such as chlorophylls, carotenoids (e.g., β-carotene, astaxanthin), and phycobiliproteins, which contribute to antioxidant activity [40]. The colors of microalgae are a result of pigments produced in the chloroplasts, such as chlorophylls and phycobiliproteins [41]. These colors are used in many fields, from food coloring to the environmental sector. Table 1 provides a summary of bioactive compounds derived from microalgae, as well as their applications and health benefits. Figure 1 shows the metabolites produced by microalgae.

Microalgae have a wide range of applications across science, industry, and environmental management due to their rapid growth, high productivity, and biochemical diversity [56]. One of the most prominent applications is in biofuel production. Certain species of microalgae can accumulate large amounts of lipids, which can be converted into biodiesel [57]. Compared to traditional crops, they require less land and can grow in non-arable environments, making them a promising renewable energy source. In the food and nutrition sector, microalgae are used as dietary supplements because they are rich in proteins, vitamins, and essential fatty acids [58,59]. For instance, spirulina and chlorella are widely consumed for their health benefits and are often marketed as superfoods. Furthermore, algal-based omega-3 fatty acids are becoming increasingly integrated into daily human consumption due to their vegan and sustainable nature [60]. Microalgae also play an important role in wastewater treatment. They can absorb nutrients such as nitrogen and phosphorus from wastewater, helping to reduce pollution while simultaneously producing biomass that can be reused in other applications [61]. In the pharmaceutical and cosmetic industries, microalgae are valued for their bioactive compounds, including antioxidants, pigments, and anti-inflammatory substances. These compounds are used in skincare products and are being studied for potential therapeutic uses [62]. Another key application is in carbon capture. Through photosynthesis, microalgae absorb carbon dioxide, which makes them useful in efforts to mitigate climate change by reducing greenhouse gas concentrations in the atmosphere [63]. Additionally, microalgae are used in aquaculture and agriculture as feed for fish and other marine organisms, as well as biofertilizers that improve soil quality and plant growth [64]. The economic value of microalgae stems not only from the biomass itself, but also from the high-value-added by-products obtained during or after production. Therefore, the modern microalgae industry is evaluated using a multi-product biorefinery model [65]. Given sustainable food systems, carbon-neutral production targets, and the increasing demand for natural products, microalgae technologies are expected to become even more important in the fields of biotechnology and the green economy in the coming years.

### Microalgal By-Products and Residual Biomass

The production processes of pigments, lipids, proteins, biofuels and other high-value compounds within the scope of microalgal biotechnology generate significant amounts of byproducts or residual biomass [66]. While for many years these residues were considered only waste material, today, with the development of sustainability and circular bioeconomy approaches, they are recognized as a valuable bioresource. Microalgal residual biomass, being rich in carbohydrates, proteins, minerals, cell wall components, and various bioactive compounds, can be used in different industrial applications [67]. Microalgal byproducts can be utilized in areas such as animal feed, organic fertilizers, biogas production, bioplastic raw materials, and agricultural applications [68]. Furthermore, it is known that the nutritional value of microalgal byproducts is increased, and they become more bioavailable through fermentation processes. In particular, the reuse of microalgal residue biomass is considered an environmentally friendly biotechnological approach, especially in terms of sustainable energy production and environmental waste management [69]. Furthermore, recent studies increasingly show that microalgal by-products have potential applications not only in energy production but also in the functional food, cosmetics, and pharmaceutical sectors [70]. The potential for use in these sectors is quite high because the health-related aspects of microalgae biomass are well known. Microalgae metabolites can be added to cream formulations in the cosmetics industry to improve and protect skin health. Efficient utilization of by-products and residual biomass stands out as one of the key strategies for enhancing the economic sustainability of microalgae-based production systems [71].

## 3. The Biological Basis of Microalgae Fermentation

Fermentation is a biological process in which a microorganism converts carbohydrates, usually starch or sugar, into fundamental components such as alcohols, organic acids, and gases [72]. Fermentation, one of the oldest and most widely used food preservation and processing methods in the world, dates back to before 10,000 BC [73]. Over time, the development of fermentation technology has enabled its practical applications to spread to other fields, such as pharmaceuticals/chemicals, biotechnology and nutrition. The most common groups of microorganisms involved in fermentation processes include bacteria, yeasts, and molds that produce enzymes that catalyze the fermentation process [74,75]. Biochemical changes carried out by microorganisms during fermentation improve the functional value and nutritional properties of the products and directly contribute to the release or alteration of bioactive compounds [76].

**Figure 2:**
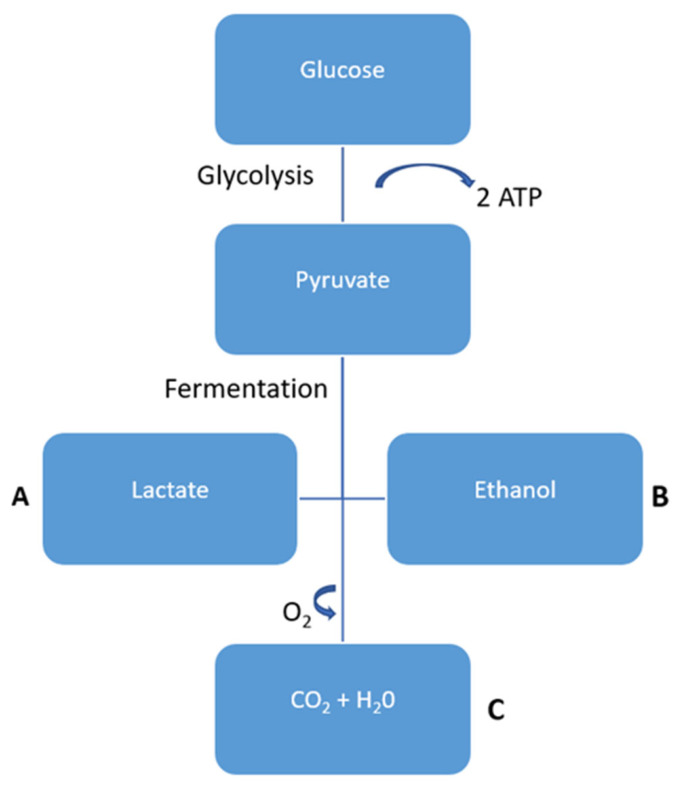
Cellular metabolic pathways: (A) lactic acid fermentation, (B) alcohol fermentation, (C) aerobic respiration.

In general, fermentation refers to an anaerobic process that usually does not require oxygen; however, oxygen may be present in some fermentation process. In the presence of oxygen, pyruvate is broken down into carbon dioxide (CO_2_) through cellular respiration, hence creating ATP molecules to provide cellular energy. Even so, if oxygen is absent or at a low level, pyruvate utilization can continue through the fermentation process [77,78]. Two common fermentation types are lactic acid fermentation and alcohol fermentation. Lactic acid fermentation is a type of fermentation in which glucose is converted into lactic acid (lactate) by lactic acid bacteria in an anaerobic environment [79]. Alcohol fermentation is defined as the breakdown of sugars into ethyl alcohol and carbon dioxide by bacteria or yeast species in an anaerobic environment [80]. In addition to ethyl alcohol and carbon dioxide, substances such as acetaldehyde, mixed acid, and acetic acid are also formed by alcohol fermentation [81]. These products, which form as a result of fermentation and cellular respiration, are shown in Figure 2.

Microalgae have become a significant research topic in fermentation technologies in recent years due to their high biomass production capacity, rich biochemical content, and environmental sustainability advantages [82]. As photosynthetic microorganisms, microalgae produce biomass rich in carbohydrates, proteins, lipids, vitamins, and pigments [83]. Thanks to these properties, they are utilized in many different areas, from biofuel production to functional foods, animal feed, and pharmaceutical products [84]. In industrial fermentation, the most commonly used microorganisms are LAB, *Bacillus* spp., yeasts, and mold fungi [85]. During the fermentation process, microorganisms obtain the nutrients and energy they need to survive by metabolizing the compounds around them, using the heat released in the process. Microorganisms have different proteolytic enzymes. Therefore, the functionality of the resulting hydrolysates varies depending on the microorganism used [86]. The type of carbon source used in fermentation processes and the concentration directly affect the growth of microorganisms and metabolite production. While glucose is one of the most commonly used substrates, alternative carbon sources such as acetate and glycerol are also widely preferred [87,88]. In addition, hydrolyzates of agricultural and industrial wastes are considered a low-cost substrate source. This situation increases the importance of fermented microalgae in terms of sustainable biotechnological applications [89].

One of the important effects of fermentation on microalgal substrates is the degradation of the microalgal cell wall. Many microalgal species possess resistant cell walls composed of cellulose, hemicellulose, pectin-like polymers, glycoproteins, or sporopollenin-like compounds [90]. During fermentation, various hydrolytic enzymes and organic acids produced by lactic acid production cause the weakening or partial degradation of these structural components. The acidification resulting from lactic acid production increases the permeability of the cell wall, facilitating the release of intracellular nutrients and biologically active compounds [91]. Particularly in species with rigid cell walls, such as chlorella and *Schizochytrium* sp., lactic acid fermentation has been reported to significantly improve nutrient extraction and digestibility [92,93]. Another important outcome of LAB fermentation is the breakdown and biotransformation of macromolecules [94]. Proteins in microalgal biomass can be hydrolyzed into peptides and free amino acids thanks to microbial proteolytic activity [95]. These small molecules are generally more easily digestible and may possess additional biological activities, such as antioxidant, antihypertensive, or antimicrobial properties [96]. Similarly, complex polysaccharides can be broken down into oligosaccharides with prebiotic potential [97]. Lipid metabolism can also change during fermentation, contributing to the release of fatty acids and altering the lipid profile [98].

LAB can also promote the formation of new biologically active compounds from fermented microalgae. During their metabolic activities, LAB can synthesize organic acids, bacteriocins, exopolysaccharides, vitamins, and antioxidant metabolites, thereby increasing the functional value of fermented microalgae products [99]. Some LAB strains can also produce biologically active peptides through the enzymatic hydrolysis of microalgae proteins [100]. These newly formed compounds can contribute to increased antioxidant capacity, antimicrobial activity, immune system modulation, and support of gut health [101,102]. The fermentation process also significantly contributes to the improvement of sensory characteristics. One of the most important limitations of microalgal biomass in food applications is the presence of strong earthy, fishy, or marine-like tastes and odors [103]. LAB fermentation helps reduce volatile compounds responsible for these undesirable sensory characteristics while simultaneously promoting the formation of desirable aroma compounds such as organic acids, esters, aldehydes, and other aromatic metabolites [104]. Acidification also contributes to the formation of a fresher and more acceptable taste profile. Thus, fermentation increases consumer acceptance and expands the potential of microalgae in fermented beverages, dairy alternatives, snacks, and other food products [105]. In addition to nutritional and sensory improvements, LAB fermentation can also enhance the safety and preservability of microalgal substrates. Lactic acid production lowers the pH of the medium, suppressing the growth of spoilage-causing and pathogenic microorganisms. Furthermore, antimicrobial compounds such as bacteriocins produced by some LAB strains can further improve microbial stability and shelf life. These features support the development of naturally preserved microalgal products without the need for synthetic additives [106,107].

## 4. The Improvement in Nutritional and Functional Characteristics of Fermented Microalgae

Fermented microalgae represent a highly promising area within biotechnology, combining the metabolic versatility of microalgal systems with the transformative capabilities of microbial fermentation. Microalgae are known for their rapid growth rates, suitability for sustainable production, ability to adapt to environmental conditions, and high protein, carbohydrate, and fat content [108]. Furthermore, one of the main characteristics of microalgae is their ability to produce bioactive compounds with potential benefits for human health [109]. However, their direct use in food, feed, and pharmaceutical applications is often limited by factors such as rigid cell walls, low digestibility, and undesirable sensory properties [110]. Fermentation offers a powerful strategy to overcome these limitations while enhancing the functional value of microalgal biomass. The fermentation of microalgae involves the use of a range of heterotrophic microorganisms [111]. These typically include lactic acid bacteria, yeasts, and filamentous fungi. During this process, microbial enzymes degrade complex polysaccharides and cell wall components, leading to increased bioavailability of intracellular nutrients such as proteins, lipids, vitamins, and pigments [112]. For example, lactic acid fermentation using *Lactobacillus* species can significantly improve protein digestibility and enhance nutrient-friendly factors. Moreover, fermentation can result in the synthesis of novel metabolites, including organic acids, bioactive peptides, and antioxidant compounds, thereby fortifying the nutritional and therapeutic potential of the biomass [113]. The nutritional and functional properties of fermented microalgae are enriched with proteins/peptides, lipids, polysaccharides, and antioxidants.

### 4.1. Antioxidant Substances

Fermented microalgae have been extensively researched in recent years in the fields of functional foods and nutraceuticals due to their high antioxidant potential. Significant increases in the biological activity of microalgal species, particularly spirulina and *Chlorella vulgaris*, have been reported after fermentation [114,115]. During the fermentation process, microorganisms break down the microalgal cell wall, increasing the bioavailability of phenolic compounds, carotenoids, and pigments. The antioxidant effect of microalgae is primarily due to carotenoids, phycocyanins, phenolic compounds, tocopherols, and polysaccharides. In a study by Choi et al., it was observed that the enhanced total antioxidant capacity and beta-carotene profile of A. maxima fermented by *L. plantarum* contributed to the apparent higher level of brain-derived neuroprotective factor compared to its unprocessed control [116]. A study published in 2025 by Tomassi et al. reported that the total polyphenol content in fermented *Chlorella vulgaris* biomass approximately doubled, and a significant increase in ORAC antioxidant capacity occurred [117]. The same study also indicated that fermentation contributed to a reduction in oxidative stress in human erythrocytes. It is believed that these compounds are released and enriched through fermentation. These compounds can neutralize free radicals, reduce cellular damage, and suppress the formation of reactive oxygen species. Oxidative stress, in particular, is known to be associated with aging, cardiovascular diseases, neurodegenerative diseases, and inflammation. However, a significant portion of current studies are at the in vitro or animal model level. Clinical trials in humans are still limited. Therefore, more clinical research is needed to clarify the long-term effects of fermented microalgae antioxidant properties on health [118,119].

### 4.2. Proteins/Peptides

Fermentation is a crucial process that biochemically transforms the protein structure of microalgae. During this process, proteolytic enzymes secreted by microorganisms can break down microalgal proteins into smaller peptides and free amino acids, altering both their nutritional value and biological activity. During fermentation, proteins are converted into oligopeptides, oligopeptides into short-chain bioactive peptides, and some peptides into free amino acids [120]. Protease enzymes found in microorganisms such as LAB, yeast and Bacillus species play a crucial role in these converts. Furthermore, the acidic environment created during fermentation can lead to changes in the secondary and tertiary structures of proteins [121]. The breakdown of hydrogen bonds and protein denaturation result in a looser protein structure, making it more susceptible to enzymatic degradation. One of the most important outcomes of the fermentation process is the formation of short-chain peptides exhibiting biological activity [122]. These peptides can display antioxidant, antihypertensive, antimicrobial, and anti-inflammatory properties. In particular, peptides containing hydrophobic amino acids have been reported to neutralize free radicals, chelate metal ions, and reduce oxidative stress. Studies on fermented spirulina samples have shown that low-molecular-weight peptides increase the DPPH radical scavenging capacity [123]. Furthermore, it has been suggested that some peptides may exhibit angiotensin-converting enzyme (ACE) inhibitor activity, thus producing an antihypertensive effect. In the study conducted by Uzlasır et al., it was determined that fermentation increases the ACE inhibitor potential and that samples fermented with *Lactiplantibacillus plantarum* showed the highest ACE effects [124]. During fermentation, the hydrolysis of proteins results in an increase in the amount of free amino acids. An increase is observed in amino acids such as glutamic acid, leucine, valine, tyrosine, and phenylalanine, in particular. These changes are thought to affect taste formation, biological activity, digestibility, and functional properties. Increased glutamic acid supports umami taste formation, while aromatic amino acids are reported to contribute to antioxidant capacity [125]. However, in long-term fermentation processes, a decrease in total protein content may occur due to the metabolism of some amino acids by microorganisms. Modifications in the protein structure of fermented microalgae also affect their technological and functional properties. After fermentation, solubility can increase, emulsifying properties can improve, water retention capacity can increase, and digestibility can be improved. Therefore, fermented microalgae proteins have the potential to be used in functional beverages, sports nutrition, protein supplements, and alternative protein-based products [126].

### 4.3. Lipids

Microalgae are among the most important biotechnological resources due to their high content of polyunsaturated fatty acids (PUFAs), phospholipids, glycolipids, and sterols. Lipase and esterase enzymes secreted by microorganisms during fermentation can cause the breakdown of complex lipids and the formation of free fatty acids [127]. Furthermore, significant changes in the fatty acid profile can occur as a result of oxidative reactions, biohydrogenation, and lipid rearrangements. Lipase enzymes secreted by microorganisms during fermentation can break down complex lipid structures. In this process, triglycerides can be converted into diglycerides and monoglycerides, and ester-linked fatty acids can be converted into free fatty acids. These hydrolytic reactions can increase the bioavailability of lipids [128]. In particular, the disrupt of the microalgal cell wall facilitates the release of lipid droplets. The low pH environment created during fermentation can also cause changes in membrane structure, facilitating lipid extraction. Changes in lipid structure can directly affect the functional properties of fermented microalgae. Specifically, increases in omega-3 bioavailability, improved digestibility, and variations in emulsifying properties may occur [129].

### 4.4. Polysaccharides

Microalgae are among the most important sources of biofunctional compounds due to the sulfated polysaccharides, β-glucans, heteropolysaccharides, and cell wall carbohydrates found in their structures [130]. During fermentation, hydrolytic enzymes secreted by microorganisms can disrupt polysaccharide chains, leading to the formation of lower-molecular-weight oligosaccharides. These transformations can result in changes in antioxidant, prebiotic, immunomodulatory, and antimicrobial activities. Enzymes such as amylase, cellulase, xylanase, and β-glucosidase, secreted by microorganisms during fermentation, are capable of breaking down polysaccharide chains [131]. Since microalgal cell walls have a highly complex structure, fermentation facilitates their disruption and enriches the release of polysaccharides [132]. In particular, it is known that carbohydrate metabolism, active in fermentation, is carried out with lactic acid bacteria. Low-molecular-weight oligosaccharides, formed as a result of the hydrolysis of polysaccharides, can exhibit prebiotic properties [133]. These compounds support the gut microbiota, promote the growth of Lactobacillus and Bifidobacterium, and increase the production of short-chain fatty acids [134]. Some oligosaccharides obtained from fermented microalgae have been reported to support gut health and reduce inflammation [135]. In their study, Xie and Cheong showed that oligosaccharides derived from algae are fermented by gut bacteria to form short-chain fatty acids (SCFAs), which are associated with prebiotic and anti-inflammatory effects [136]. Hyrslova et al. investigated the health effects of combining *Chlorella vulgaris*, known for its high EPS content, with *Bifidobacterium animalis* subsp. *lactis* BB-1. The study showed a reduction in triglyceride levels in the serum, liver, and heart of mice. The same study claimed that incorporating *C. vulgaris* along with bifidobacteria into functional food products may help maintain the viability of the bifidobacteria [137].

### 4.5. Carotenoids

Carotenoids are lipophilic pigments synthesized by photosynthetic organisms and known for their antioxidant properties. Microalgae are important natural producers of biologically valuable carotenoids such as β-carotene, lutein, zeaxanthin, astaxanthin, and fucoxanthin [138]. Fermentation is a process involving the biochemical transformation of organic substrates through microorganisms and can alter the functional properties of microalgal biomass. In recent years, numerous studies have been conducted on microalgal fermentation, particularly on its potential to increase carotenoid production and improve bioavailability. Fermentation alters not only the total amount of carotenoids but also their composition [139,140]. It has been observed that some carotenoids are converted into derivative forms as a result of enzymatic oxidation and isomerization reactions. These transformations are considered part of the stress adaptation mechanisms of microalgae. The breakdown of the microalgal cell wall during fermentation facilitates the release of carotenoids. This provides a significant advantage, particularly in terms of absorption by the human digestive system. Literature data show that carotenoid bioavailability is significantly increased in fermented microalgal products [141].

Carotenoids are powerful free radical scavengers. It has been reported that their antioxidant capacity increases at controlled fermentation rates, but decreases due to oxidative degradation during prolonged or uncontrolled fermentation [142].

**Figure 3:**
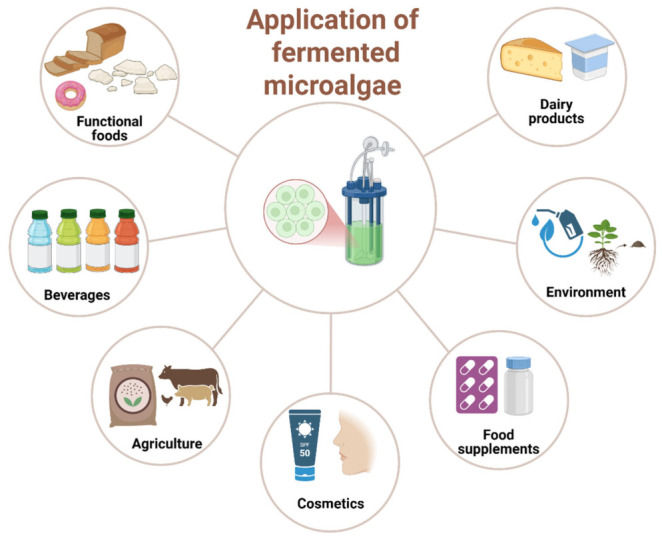
Current applications of fermented algae.

As microalgae acquire increasingly remarkable properties, the applications of fermented microalgae are expanding across many different sectors. Fermented algae have received more attention in the last decade and have been the subject of numerous scientific studies. In the food industry, fermented microalgal products are being developed as functional ingredients, offering enhanced flavor profiles and improved nutritional quality [143,144]. To enhance the nutritional content of dairy products, particularly different types of cheese, fermented algae can be added. By combining these two food forms, it is possible to produce nutritious foods with high nutritional content [145]. They can also be added to yogurts, beverages, and snacks for probiotics, vitamins, and antioxidants [146]. In the pharmaceutical and nutraceutical fields, fermented microalgae are being investigated for their antioxidant, anti-inflammatory, and antimicrobial properties [147]. Their inclusion in health supplement formulations has increased demand. The various field applications of fermented microalgae are shown in Figure 3.

Numerous studies on microalgal fermentation have been conducted in recent years. Many of these studies focus on cyanobacteria, particularly *Arthrospira* species. *Chlorella* is the next most commonly studied species. The microorganisms used in fermentation are generally LAB species and yeasts. Studies using a combination of these two microorganisms also exist. Research has generally focused on improving the antioxidant, total phenolic, and bioactive substance content of fermented microalgae. These efforts have been demonstrated in method development and innovative biotechnological approaches. The details of the studies are given in Table 2. Nicolotti et al. found that lactic acid fermentation significantly improved the aroma profile of *Chlorella vulgaris* by reducing aldehydes [148]. In another study, Martelli et al. showed that various types of LAB and yeast-fermented microalgae can produce bioactive peptides with potential metabolic health benefits [149]. Another study determined that mixed fermentation systems enhanced both the digestibility and the sensory quality of microalgal biomass [150]. Verni et al. demonstrated that the products obtained by using spirulina and chlorella in LAB culture medium showed positive effects in terms of antioxidant, antimicrobial and taste properties [151]. To summarize, the fermented algae industry is expected to grow in the coming years thanks to advances in downstream methods, the increase in application areas, and the interaction between innovative technologies. With the ever-increasing global population, the tendency towards healthy eating and the human goal of longevity, the development of fermentation-based healthy foods will become even more important. Furthermore, the resulting by-products and residual biomass will be integrated with biorefinery systems to develop innovative solutions.

## 5. Health Aspects of Fermented Microalgae and Their Biomass

**Figure 4:**
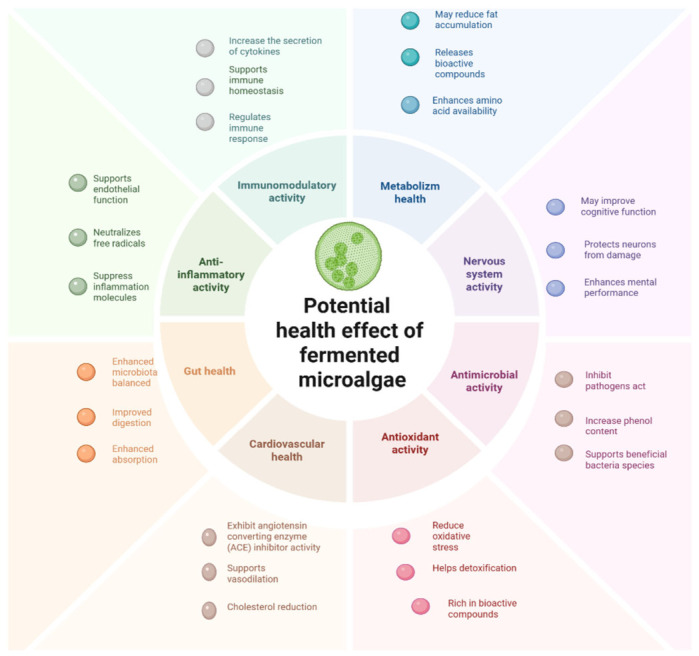
Potential health effects of fermented algae.

Potential health effects are associated with the consumption of fermented algae, which possess peculiar nutritional and functional properties. Current studies have demonstrate that bioactive compounds in microalgae play a crucial role in promoting health [160]. Fermented algae have many potential health benefits, which are shown in Figure 4. The interaction of microalgae with the fermentation process makes it possible to release complex molecules, enrich secondary metabolites, and obtain biocompatible products. Fermentation also improves nutritional value, ensures sustainable production, and is promising in other application areas. Fermented microalgae exhibit improved protein digestibility, enhanced mineral absorption, and greater accessibility of essential fatty acids. Even though studies have generally focused on antioxidant activity, fermented algae contribute to many different systems, from cardiovascular health to metabolic health [161].

Fermented microalgae demonstrate remarkable anti-inflammatory properties. Bioactive peptides produced through proteolysis during fermentation can modulate inflammatory pathways by influencing cytokine production and immune cell activity [162]. Furthermore, certain fermentation-derived metabolites, including short-chain organic acids and fatty acid derivatives, have been shown to exert immunomodulatory effects. Fermented algae suppress inflammation molecules and help support endothelial function. These properties suggest potential applications in managing inflammatory conditions and supporting immune health [163].

Emerging evidence also suggests that fermented microalgae may play a role in metabolic health. Microalgae are naturally rich in omega-3 fatty acids, such as eicosapentaenoic acid (EPA), which are associated with lipid regulation and cardiovascular health [164]. Angiotensin-converting enzyme (ACE) is an indicator of hypertension activity, and fermented algae have been found to affect this enzyme as well. Fermentation can enhance the bioaccessibility of these lipids and generate additional metabolites that influence lipid metabolism [165]. Experimental studies, particularly in animal models and cell culture, have demonstrated reductions in lipid accumulation and improvements in metabolic parameters following the consumption of fermented microalgal products [166]. Fermented algae can reduce fat accumulation and increase the biocompatibility of amino acids. Various peptide fractions obtained from *Spirulina platensis* fermented with a mixture of multiple microorganisms significantly contribute to an enhancement in immunomodulatory activities. Furthermore, fermented spirulina increases lymphocyte proliferation compared to unfermented spirulina [167]. Fermented algae support immune homeostasis and help regulate immune responses. Current literature indicates that the fermentation process leads to an enhancement in bioactive components associated with the immunomodulatory effect of microalgae.

The antioxidant activity of fermented microalgae has attracted considerable interest due to the significant enhancement in bioactive compounds and redox-related metabolites achieved through microbial biotransformation. Microalgae are naturally rich in antioxidant molecules, including carotenoids, chlorophylls, phenolic compounds, vitamins, and polyunsaturated fatty acids (PUFAs) [168]. However, the bioavailability and functional efficacy of these compounds are often limited in non-fermented biomass. Fermentation has been demonstrated to substantially improve both the quantity and activity of antioxidant constituents [169]. The antioxidant activity of fermented microalgae is commonly evaluated using in vitro assays such as DPPH, ABTS, FRAP, and ORAC [170]. Studies consistently report significant increases in antioxidant activity after fermentation, often correlating with elevated total phenolic content and peptide formation [171]. Furthermore, the altered pigment profile during fermentation is also important for antioxidant activity. Carotenoids and chlorophyll derivatives obtained from microalgae, such as β-carotene, astaxanthin, and lutein, can increase their antioxidant activity by undergoing structural changes during fermentation [172]. These pigments are thought to scavenge free radicals, thus protecting cellular components from oxidative damage. For example, lactic acid fermentation of *Chlorella vulgaris* has been shown to increase DPPH radical scavenging activity and reducing power, while also enhancing polyphenol levels [173]. Similarly, fermented *Arthrospira platensis* exhibits improved antioxidant capacity due to the release of phycocyanin and other pigment–protein complexes [174]. The enhanced antioxidant activity of fermented microalgae is particularly relevant in the prevention and management of oxidative stress-related diseases, including cardiovascular diseases, neurodegenerative disorders, diabetes, and cancer [175]. These studies are generally conducted in cell cultures and animal models. Clinical trials are currently insufficient. By reducing oxidative stress, these products may help protect lipids, proteins, and DNA from damage, thereby supporting cellular homeostasis and overall health. With the fermentation process, microbial metabolism can convert phenolic precursors into more active derivatives with higher radical scavenging capacity. Additionally, proteolytic activity generates bioactive peptides with antioxidant properties capable of neutralizing reactive oxygen species (ROS) and chelating metal ions [176]. These peptides contribute significantly to the overall antioxidant capacity of fermented microalgal products. In their study, Ryu et al. showed that *P. lutheri* microalgae fermented with *C. rugopelliculosa* have the ability to scavenge free radicals (0.01–1000 g/mL of fermented microalgae), especially hydroxyl radicals, in a dose-dependent manner [177]. This antioxidant ability has been largely attributed to the production of bioactive peptides and amino acids. In another study, Qian et al. fermented *Pavlova lutheri* (FMP) Butcher microalgae with *Hansenula polymorpha* yeast [178]. The fermented preparation was tested for antioxidant activities, including lipid peroxidation inhibitory activity, free radical scavenging activity, inhibition of reactive oxygen species (ROS) in mouse macrophages (RAW264.7 cells), and inhibition of myeloperoxidase (MPO) activity in human myeloid cells (HL60). FMP showed the highest antioxidant activity in free radical scavenging, intracellular ROS inhibition, and inhibition of MPO activity. The MTT [3-(4,5-dimethyl-2-yl)-2,5-diphenyltetrazolium bromide] test found no cytotoxicity in mouse macrophages (RAW264.7 cells), human myeloid cells (HL60), and the human fetal lung fibroblast cell line (MRC-5). Grover et al. reported that bioactive peptides have antiproliferative effects against Caco-2 cells [179]. C-phycocyanine, a pigment obtained by extraction of spirulina, had a strong immunomodulatory effect and no cytotoxic effect in a study on an animal model. In another study, Liu et al. established that, within an MTT assay after UVB irradiation, HaCa T cells (skin cells) treated with different LAB species fermented with *A. platensis* demonstrated protective effects against UVB radiation [180].

Fermented microalgae are increasingly recognized for their potential to support gut health, owing to their combined prebiotic, probiotic, and postbiotic effects [181]. Examples of commonly used microorganisms include strains of *L. plantarum*, *S. thermophilus* and *L*. *rhamnosus*. Microalgae such as *Arthrospira platensis* and *Chlorella vulgaris* contain polysaccharides, proteins, and bioactive compounds that can beneficially interact with the gastrointestinal microbiota [182]. However, in their biomass form, many of these compounds are not fully accessible due to the presence of rigid cell walls [183]. Fermentation amplifies their functionality by transforming both the structure and composition of the biomass. Fermented microalgae can aid the digestive system, providing a more balanced microbiota and improved absorption. Fermented microalgae can act as probiotic carriers when fermentation is conducted using live beneficial microorganisms, particularly lactic acid bacteria. These microorganisms can survive to colonize the gut, where they exert health-promoting effects, including the inhibition of competitive pathogenic bacteria, enhancement of mucosal barrier function, and modulation of immune responses [184]. Fermented microalgae can be marketed for use in functional food products. These products have significant market size and are attracting consumer interest. Fermented microalgae also lead to the release of compounds such as short-chain fatty acid (SCFA) precursors and bioactive peptides [185]. Additionally, microalgae biomass also represents a promising feedstock for the sustainable production of a wide range of volatile organic compounds (VOCs) for food applications [186]. These metabolites contribute to gut health by lowering intestinal pH, inhibiting the growth of harmful microorganisms, and acting as signaling molecules that regulate host metabolism and immune function [187]. Fermented algae have also been found to exhibit antimicrobial effects. The proteolytic activity of lactic acid bacteria involved in fermentation leads to the formation of small peptide groups that showing antimicrobial activity. Tolpeznikaite et al. determined that the antibacterial effect of fermented spirulina products was much better expressed against Gram-positive bacteria than against Gram-negative bacteria [188]. In addition, the strong antimicrobial activity of fermented spirulina against *S. aureus* was reported.

Fermented microalgae are thought to have potential effects on the nervous system, particularly through mechanisms such as the gut–brain axis, antioxidant activity, anti-inflammatory effects, and neuroactive compound production. Although research in this area is still very new, current evidence suggests that fermentation may enhance the neuroprotective properties of microalgal biomass. It is thought to have positive contributions to mental and cognitive health [189].

Overall, studies show that fermented microalgae possess antioxidant effects, metabolic properties, benefits for the intestinal system, and anti-inflammatory effects. The fermentation process can enhance the health effects of microalgae by increasing their bioavailability. They are considered promising biotechnological products, particularly in the fields of functional foods and nutraceuticals. However, much of the current evidence is based on in vitro and animal studies; clinical human trials are still insufficient. The major limitations that fermented microalgae need to overcome include limited clinical studies, lack of standardization, variability in fermentation conditions, the fact that not all microalgae species show the same effect, dose uncertainty, and limited safety data. However, studies on fermented microalgae have increased in recent years, and it is thought that these limitations will be overcome in the future.

## 6. Conclusions and Future Perspectives

Microalgae, one of the oldest known types of living organisms, have become increasingly popular in recent years because of their nutritional and health-promoting properties. Their rich nutritional value, potential health benefits, and integration into many different industries have enabled them to find a place in different markets. Although fermentation is a widely used technology, the concept of fermented microalgae is relatively novel. Fermentation can enhance the nutritional value and health benefits of microalgae. Fermentation increases the bioactive components that may offer health benefits, improves digestibility, and fortifies the texture and flavor of microalgae. With the increasing interest in functional foods and healthy eating, it is thought that fermented microalgae will be used more frequently in final product formulations. Particularly due to their potential health benefits, they can be used as nutritional supplements or functional foods. In conclusion, the fermentation of microalgae represents a synergistic approach that fosters the nutritional, functional, and economic value of microalgal biomass. Continued research into microbial interactions, metabolic pathways, and bioprocess engineering will be essential to fully realize the potential of this innovative biotechnological approach. On the other hand, in vivo studies on fermented microalgae are insufficient. In the near future, bioavailability and biosafety studies require further research in human or animal models. Once these conditions are handled, fermented microalgae may gain a larger market share.

## Figures and Tables

**Table 1:** Bioactive compounds from microalgae, their applications, and their health benefits.

Product	Key Compounds	Microalgae Sources	Applications	Health Benefits	Reference
Pigments	Chlorophyll, β-carotene, astaxanthin, lutein	*Dunaliella salina*, *Haematococcus* *pluvialis*, *Spirulina maxima*	Cosmetics, food coloring, nutraceuticals	Antioxidant, eye health, skin protection, anti-aging	[40,41,42]
Proteins & Peptides	Essential amino acids, bioactive peptides	*Arthrospira platensis*	Nutraceuticals, functional foods, supplements	Muscle growth, antihypertensive, antioxidant, antimicrobial	[43,44]
Lipids & fatty acids	EPA, DHA (omega-3)	*Nannochloropsis* *oculata*, *Schizochytrium* sp., *Crypthecodinium cohnii*, *Isochrysis galbana*	Dietary supplements, pharmaceuticals, infant formulas	Heart health, brain function, anti-inflammatory, antioxidant	[45,46,47,48]
Polysaccharides	Sulfated polysaccharides	*Porphyridium* *purpureum*, *Euglena gracilis*, *Phaeodactylum* *tricornutum*	Pharmaceuticals, functional foods	Antiviral, immune-boosting, anticoagulant, anti-inflammatory	[49,50,51]
Vitamins & minerals	Vitamin B12, D, E; provitamin A; iron; magnesium	Chlorella, spirulina	Supplements, fortified foods	Immunity support, energy metabolism, anemia prevention	[52,53]
Phenolic Compounds	Polyphenols, flavonoids	*Porphyridium* *cruentrum*, *Scenedesmus obliquus*	Pharmaceuticals, nutraceuticals	Antioxidant, anticancer potential, phenolic compounds	[54,55]

**Table 2:** Studies on fermented microalgae for various applications.

Microalgae Species	Microorganisms	Method/Focus	Fermentation Condition	Key Findings	References
*Chlorella* *vulgaris*	*L. casei,* *L. paracasei*, *L. rhamnosus*, *L. plantarum*, *L. delbrueckii* subsp. *bulgaricus*, and *Leuconostoc citreum*	Lactic acid fermentation (VOC analysis)	Time: 15 h T: 37 °C	Significant reduction in off-flavor aldehydes; increased ester compounds improving aroma	[148]
*Arthrospira platensis*	*L. casei* and *L. rhamnosus*	Pre-treated with either UV light or thermal sterilization fermentation	Time: 15 h T: 37 °C % 3 *v*/*v* inoculation	Increase in certain hydrocarbons and aldehydes after fermentation	[149]
Mixed microalgae + plant substrates	5 LAB and 5 yeast species	Mixed fermentation	Time: 18 h T: 30 °C	Improved digestibility of proteins and polyphenols	[150]
*Spirulina*	*L. plantarum*	Experimental fermentation	Time: 15 h T: 37 °C	Increased antioxidant activity and peptides	[151]
*Chlorella* *vulgaris*	*L. brevis*	Co-culture fermentation	Time: 24 h T: 30 °C	Stimulates LAB growth and metabolism	[152]
*Chlorella* *vulgaris*	*L. fermentum*, *L. rhamnosus*	Beverage fermentation	Time: 24 h T: 30 °C % 10 *v*/*v* inoculation	Increased antioxidant capacity and phenolics	[153]
*Arthrospira platensis*	*L. plantarum*	Mixed fermentation	Time: 72 h T: 37 °C	Enhanced nutraceutical properties	[154]
*Spirulina*	*L. helveticus*, *K. marxianus*	Co-culture fermentation	Agitation: 150 rpm T: 37 °C Aeration rates: 0.5–1 vvm	Improved bioactive and chemical properties	[155]
*Spirulina*	*L. acidophilus*, *K. marxianus*	Co-culture fermentation	T: 35 °C Time: 24 h 5 lg (cfu/mL) inoculation	Enhanced total flavonoid and phenol levels	[156]
*Arthrospira platensis*	LAB strains	Probiotic-based products	Agitation: 100 rpm T: 37 °C Time: 72 h	Increased antioxidants and phenolics	[157]
*Arthrospira platensis*	*L. rhamnosus*	Pilot scale fermentation	Agitation: 150 rpm T: 37 °C Time: 24 h	Increased antioxidants and strong cytotoxic effect	[158]
*Spirulina*	*L. helveticus*, *K. marxianus*	Functional fermentation	T: 37 °C Time: 48 h	Bioavailability	[159]

## Data Availability

The original contributions presented in the study are included in the article; further inquiries can be directed to the corresponding author.
